# Validating evidence for the knowledge, management and involvement of dentists in a dental approach to sickle-cell disease

**DOI:** 10.1590/1807-3107bor-2024.vol38.0026

**Published:** 2024-04-05

**Authors:** Lucia Helena da Silva Ferreira ANCILLOTTI, Mauro Henrique Nogueira Guimarães de ABREU, Angélica Maria Cupertino Lopes MARINHO, Marcia Pereira Alves dos SANTOS

**Affiliations:** (a) Universidade Federal do Rio de Janeiro – UFRJ, School of Dentistry, Department of Community and Preventive Dentistry, Rio de Janeiro, RJ, Brazil.; (b) Universidade Federal de Minas Gerais – UFMG, School of Dentistry, Department of Community and Preventive Dentistry, Belo Horizonte, MG, Brazil.; (c) Faculdade de Teologia, Filosofia e Ciências Humanas de Gamaliel – Fatefig, Tucuruí, PA, Brazil.; (d) Universidade Federal do Rio de Janeiro – UFRJ, School of Dentistry, Department of Community and Preventive Dentistry, Rio de Janeiro, RJ, Brazil.

**Keywords:** Anemia, Sickle Cell, Validation Study, Oral Health

## Abstract

This study validated the content of an instrument designed to assess the knowledge, involvement (attitudes) and management (practice) of dentists relative to sickle-cell disease (KAPD-SCD). The instrument consisted of five domains composed of a total of thirteen items: I. Dentist’s self-assessment relative to sickle-cell disease; II. Dentist’s knowledge of the repercussions of sickle-cell disease on the stomatognathic system; III. Dentist’s knowledge of the complications of sickle-cell disease in the stomatognathic system; IV. Dentist’s knowledge concerning the dental management of sickle-cell disease patients; and V. Dentist’s involvement in an approach to sickle-cell disease. Twelve experts assigned scores to each item of the instrument. The criteria were clarity, understanding and appropriateness, leaving open fields for comments. Descriptive and content analyses of the data were made. Each expert analyzed 39 assessment units. The percentages considered for agreement were high (>80%), medium (70%-80%), or low (<70%), and each item was maintained or revised according to the percentage observed. There was high consensus in 74% of the assessment units (the corresponding items were maintained), medium consensus in 24% of them (the corresponding items were revised), and disagreement in 2% of them, namely as regards the “appropriateness” of item 5 (“Are there oral complications in sickle-cell disease?”), which was revised. The final version of the instrument had 16 items for different applications such as in the clinical care program, teaching program, or research program, with different cut-off scores for each application. In conclusion, the level of agreement among experts showed evidence of the content validity of the instrument.

## Introduction

Sickle cell disease (SCD) is the most common inherited genetic hemoglobinopathy in Brazil and worldwide,^1^ with high morbidity and mortality,^2^ affecting twenty million people.^2,3^ SCD is a public health problem^1^ mainly for minority groups^3^ and occurs due to the presence of a hemoglobin S mutation instead of a hemoglobin A in the red blood cells. In Brazil, the incidence of SCD is 1:1,000 live births, and an estimated number of sixty thousand people with SCD^4^ representing a global burden of disease from non-communicable chronic diseases (NCD).^2^

The S mutation of hemoglobin alters the physiology and blood tissue rheology of red blood cells during transportation of respiratory gases, leading to a wide range of clinical repercussions in organisms modulated by intense pain^5^ as a consequence of hemolytic anemia and vaso-occlusive crises. Pale mucosa, jaundice, dental opacities, and abnormalities in the maxilla and mandibular bone trabeculae occur in the stomatognathic system.^6^ Infections are recurrent, with a four-hundred-fold increase in risk.^7^ The higher prevalence of bone infections and neuropathy, notably in the craniofacial complex^8^ are common complications of SCD.^5^ Moreover, people with SCD are more likely to experience oral health concerns.^9^

Dentists and the oral health teams of the family health strategy showed a lack of knowledge about SCD and its management.^10^ Although dental students and trained dentists recognized the importance of SCD, their self-assessment revealed inadequate knowledge about the disease. They admitted the need to include the subject in undergraduate curricula and scientific meetings.^11^ Knowledge acquired by health professionals improves the quality of care and management of the disease, due to better understanding of the social, care-related, environmental, biological, and scientific dimensions of SCD.^12^

Undoubtedly, there has been a significant increase in the availability of assessment scales and/or questionnaires;^13^ however, many of these instruments do not carry adequate evidence of validation.^14-16^ In the dental literature consulted, no previously validated instrument for assessing dentists´ knowledge about SCD was found. To fill this gap, the aim of this study was to validate the content of an instrument designed for assessing the knowledge, involvement (attitudes), and management (practice) of dentists regarding SCD (KAPD-SCD).

## Methodology

### Ethical aspects

The local Research Ethics Committee approved the study (Approval No. 51985321.3.0000.5257).

### Study design

**Figure 1:**
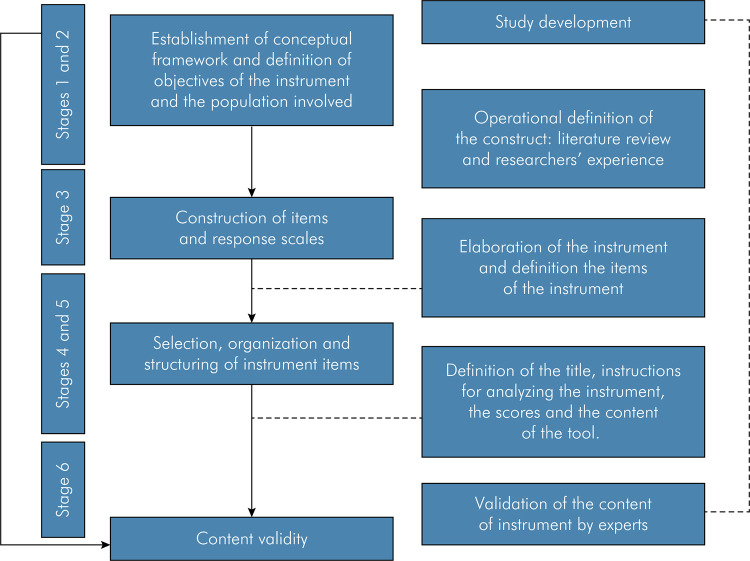
Adapted stages of this study design17 for content validity of the instrument.

This was a methodological study developed in six stages^17^ (Figure 1) between September 2021 and August 2022, based on the triangulating method. The triangulation of methods consisted of using quantitative and qualitative analysis. Therefore, quantitative analysis referred to the content of the instrument assessed by judges individually, independently, without assistance, and in a pre-defined period to obtain the committee´s agreement rate. Then, the qualitative analysis referred to the group discussion and the interactive process between researchers and committee members to clarify controversial topics and reach the final format of the instrument. All stages were in accordance with the instrument validation methodology.^18^

### Instrument development for validation of content

The authors of the present study set up the definition of the construct related to the knowledge, dental practice, and attitudes of dentists towards SCD after rigorous theoretical input based on a literature review and researchers’ experience. Subsequently, to establish a conceptual structure of the instrument, questions and their appropriate answers were elaborated based on the etiology, diagnosis, repercussions, and complications of SCD in the stomatognathic system, and on the dental practice/management, and attitudes/involvement of dentists with respect to SCD.

**Figure 2:**
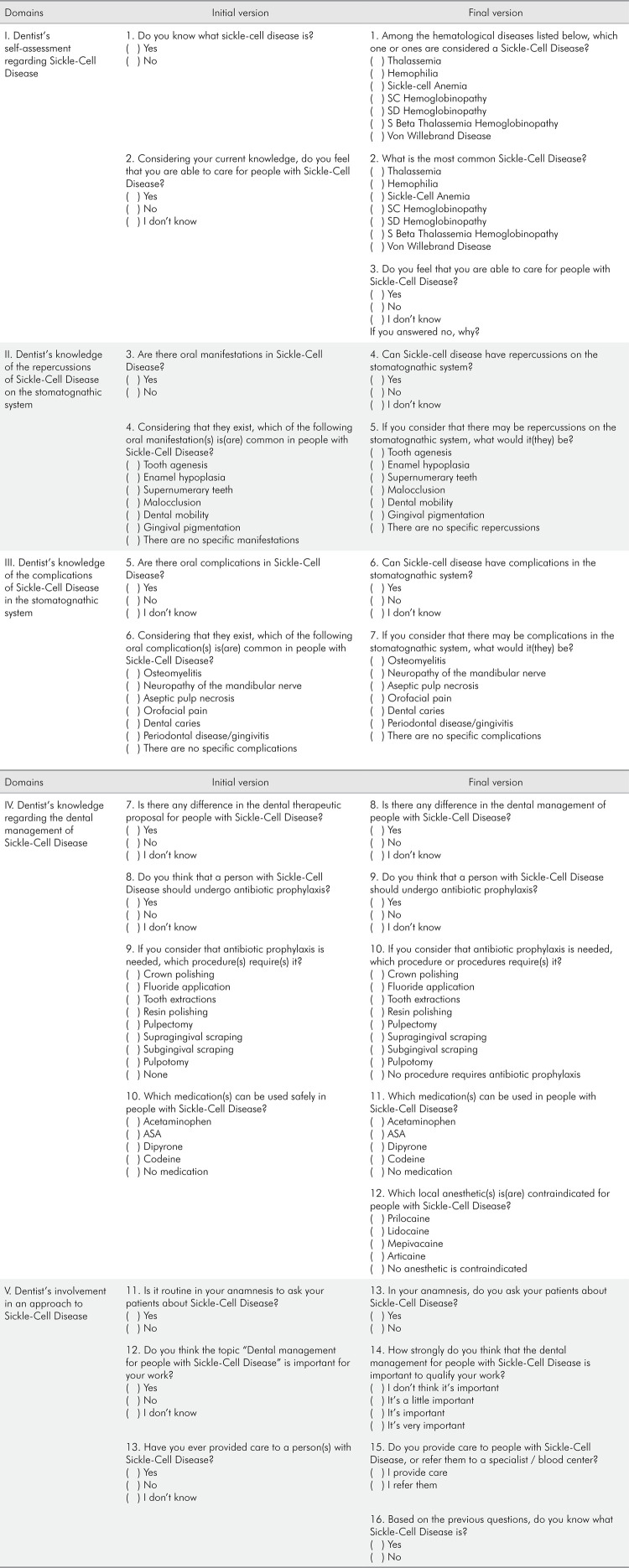
Comparison between the initial and final versions of the Instrument for Assessing the Knowledge, Involvement (Attitudes), and Management (Practice) of Dentists in a Dental Approach to Sickle-Cell Disease (KAPD-SCD). Continue.

The first version of the instrument for validation of content to assess the knowledge, involvement (attitudes), and management (practice) of dentists regarding sickle-cell disease (KAPD-SCD) consisted of 13 questions organized into five conceptual domains structured as follows: I. “Dentist’s self-assessment about SCD” (questions 1 and 2); II. “Dentist’s knowledge about the repercussions of SCD on the stomatognathic system” (questions 3 and 4); III. “Dentist’s knowledge about the complications of SCD in the stomatognathic system” (questions 5 and 6); IV. “Dentist’s knowledge about the dental management of SCD” (questions 7 to 10); and V. “Dentist’s involvement in a dental approach to SCD” (questions 11 to 13) (Figure 2).

### Composition of the expert committee

To compose the expert committee, a convenience sample of thirteen professionals^19^received a letter of invitation sent to them individually and by name via e-mail, containing detailed information about the research and their role in the study. Twelve experts responded affirmatively to the letter of invitation in accordance with the literature^20^ that indicated from 6 to 20 experts for this stage of instrument content validation. The eligibility criteria for choosing the expert committee were being a professor or researcher working in the fields of SCD and/or psychometrics, adapting and developing research instruments, and/or being an expert in the dental care of people with special needs, and/or being a member of the Regional Dental Board serving people with special needs in their respective states. To ensure at least national representation, at least one expert had to come from each area of Brazil.

Experts who agreed to participate in the study received an electronic form made available through the Google Forms platform, consisting of three sections. The first part contained: a) an explicit permission and term of informed consent; b) a preliminary version of the instrument, with 13 items divided into five domains and structured as closed questions and measurable responses; and c) the pre-established criteria with the corresponding scores for each expert’s assignment to each item of the instrument. The experts could also fill in the blank fields for each item for all criteria.

### Content validity

#### Criteria and assessment of each item of the instrument for validation of content by the experts

The experts assessed each of the 13 items of the instrument according to the criteria of clarity, understanding, and appropriateness using dichotomous values, (0) for the absence or (1) the presence of each criterion. A score of “0” for “clarity” meant that the content was unclear, and a score of “1” meant that the content was presented in clear language and without raising any doubts; a score of “0” for “understanding” meant that the content was hard to understand, and “1”, that the content was easy to understand; a score “0” “appropriateness” meant that the response alternatives were inappropriate or did not meet the question goals, and “1”, that the response alternatives were appropriate and met the question goals. Furthermore, to assess whether the primary content items had been included, whether the content items were relevant to the instrument being evaluated, whether any additional features were necessary, whether any items needed to be removed, or whether the proposed scoring system had appropriate scores and weights, the experts were given open fields in which to include qualitative considerations in terms of the three criteria evaluated for each item of the assessment tool. After concluding their content analysis, the experts’ observations were compared with the literature. Finally, the initial version of the instrument sent to the experts also contained a section designed to assess the applicability of the questionnaire to dentists. In this section, the options for application involved a) clinical care, b) research, c) educational purposes (training), and d) other, with an open field for the expert to indicate other application(s), if deemed appropriate. In this section, experts were allowed to mark more than one alternative, and responding was not mandatory. Each expert was then asked to set a cut-off score for each of the three applications described, by marking a minimum score required for each application on a numerical visual scale ranging from 1 to 10. There was also an open field in this section for the expert to explain his/her choice of minimum score if he/she considered this to be necessary.

The experts also answered two final open questions, and explained their answers, if they considered this to be necessary: “Should there be different minimum scores for the different application options in the questionnaire?” and “Should the weight of any of the items or conceptual domains of the instrument be increased?” The experts had up to fifteen days to return the completed forms.

#### Committee’s level of agreement score and validation of the instrument content by experts

There were thirty-nine assessment units (13 items x 3 assessment criteria) assessed by each of the 12 experts, totaling 468 assessment units. Two experienced researchers evaluated the experts’ suggestions for their applicability. After the judges’ assessment, the committee’s level of agreement score was obtained using the following formula: Total number of “0 or 1” scores for each assumed criteria per question/total number of assessment units per criteria x 100.

Stability of the answers was obtained when there was at least 70% agreement among the experts’ answers.^21^ A “low consensus” was defined as a percentage of agreement lower than 70%, in which case the item involved was changed or rephrased to improve clarity, understanding or appropriateness. A “medium level of consensus” was defined as a percentage of agreement in the range of 70%–80%, in which case the item involved was revised. Finally, a “high level of consensus” was defined as a percentage of agreement higher than 80%, in which case the item involved in the initial version of the instrument remained unchanged in its final version, thus showing evidence of the validity of the score assigned.^21^

For the acceptance or rejection of the experts’ comments, the researchers considered referring to the content (clarity, understanding, and appropriateness) of the instrument. This was the purpose of the experts’ judgment at the end of the validation process; and for the instrument to be based on scientific evidence and/or the most up-to-date literature on dental approaches to people living with SCD. Two experienced researchers evaluated the experts’ suggestions with regard to their applicability according to content analysis.

## Results

**Figure 3:**
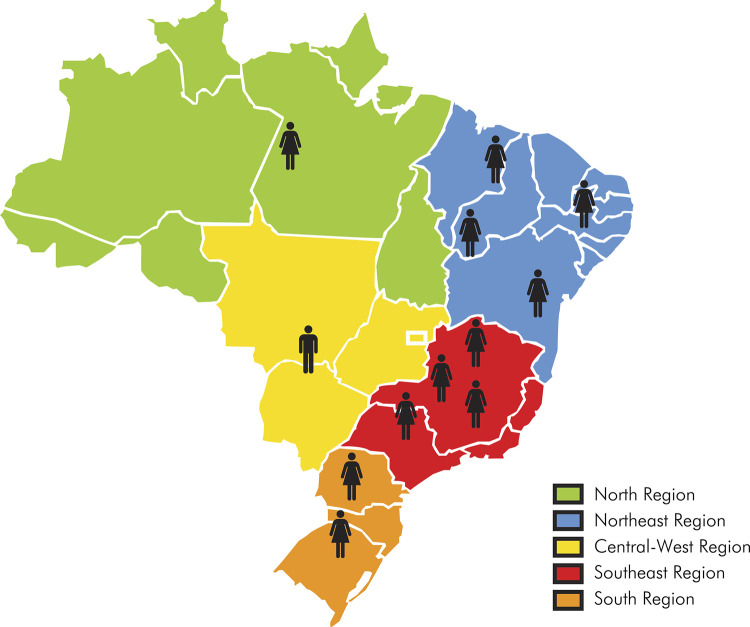
Distribution of committee experts by sex and Brazilian region.

Figure 3 shows the number of experts by Brazilian region and by sex. Ninety-two percent of the experts accepted the invitation to participate in the committee. Table exhibits the percentages of consensus among the experts. There was a high consensus in 74% of the assessment units (346/468), Medium consensus in 24% (112/468), prompting revision of the corresponding items, and lastly, there was disagreement in 2% (10/468), namely in terms of the criterion of appropriateness applied to question 5: “Are there oral complications of SCD?” (Figure 1, question 5).

**Table:** Level of consensus among experts regarding the clarity, understanding and appropriateness criteria for the 13 items of the instrument, in its initial version, divided into 5 conceptual theoretical domains.

Domain	I (%)		II (%)		III (%)		IV (%)				V (%)		
Question	1	2	3	4	5	6	7	8	9	10	11	12	13
Clarity	91.66	100.00	91.66	100.00	75.00	75.00	75.00	75.00	83.33	91.66	100.00	100.00	100.00
Understanding	91.66	100.00	91.66	100.00	75.00	75.00	83.33	75.00	100.00	91.66	100.00	100.00	100.00
Appropriateness	75.00	91.66	83.33	83.33	66.66	75.00	91.66	100.00	100.00	90.00	81.81	100.00	100.00

Red (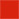): low level of consensus among specialists (<70%), the question was changed according to the pertinence of the expert’s argument; yellow (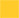): medium level of consensus among specialists (70%–80%), the question was revised after conducting a literature review; green (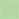): high level of consensus among experts (>80%), the question was left unchanged in the final version of the instrument.

There was medium consensus regarding appropriateness, and high consensus regarding clarity and understanding, for question 1 of domain I (“Dentist’s self-assessment regarding SCD”, questions 1 and 2). In his open comment, expert 1 said “I think it’s important to differentiate SCD from sickle-cell anemia due to the diversity of manifestations/symptoms, considering that these technical terms could prompt conceptual doubts among general practitioners, produce interpretation bias in relation to this question, and, as result, biased responses.” Other experts also highlighted that the differences in approaches to dental management were exclusive to sickle-cell anemia, and not to all other SCDs. In his comment to the same question 1, expert 2 considered that “The term SCD involves a series of conditions. The dentist will hardly know the difference between SCD and sickle-cell anemia. Perhaps it would be better to build an instrument with the aim of evaluating sickle-cell anemia, since it is the most frequent condition among those diagnosed as being SCD patients. On the other hand, if the dentist is aware that SCD includes several conditions other than sickle-cell anemia, he/she may become confused when responding to the subsequent questions. An alternative to changing the question would be to include another question after it, to check whether the responding colleague really knows what SCD is, and then confirm this in the following question.” For expert 3, “the term SCD encompasses several conditions. Even if the dentist answers that he knows what SCD is, we will not know if he really does. Many will probably mistake the term SCD for sickle-cell anemia. To check whether the dentist really understands the term SCD, a new question should be included.” The question 1 was revised and divided into two questions. The first question detailed the group of diseases conceptually covered by the term SCD in the answer alternatives. Whereas, in the question statement itself, the second question asked what the most common type of SCD was, and then included the term sickle-cell anemia among the answer alternatives.

Although there was high consensus among experts regarding question 3 (“Are there oral manifestations of SCD?”) of domain II (“Dentist’s knowledge of the repercussions of SCD on the stomatognathic system,” questions 3 and 4), one of the experts suggested changing the terminology “oral manifestations” to “repercussions on the stomatognathic system.” The researchers promptly accepted the suggestion. Based on this same rationale, the terminology “oral manifestations” in question 4 was also changed to “repercussions on the stomatognathic system.”

Questions 5 and 6 of domain III (“Dentist’s knowledge of the complications of SCD in the stomatognathic system”) were changed based on the experts’ suggestion and based on the same argument as that used for the previous domain, by replacing “oral complications” with “complications in the stomatognathic system.” There was low consensus among the experts’ responses to question 5, prompting its revision and rephrasing based on the appropriateness criterion. Hence, “Are there oral complications in SCD?” was changed to “In SCD, can there be complications in the stomatognathic system?” The greatest divergence occurred in domain III, in which there was medium to low consensus among experts due to disagreement between the terms “repercussions” in the previous domain and “complications” in domain e. To some experts, these terms could raise doubts among general practitioners, since they believed that the two terms were similar, and could be unified. Other committee members considered that some of the oral manifestations of SCD were actually complications, and vice versa. In her comments about question 5, expert 3 pointed out that “In any event, the discussion about oral manifestations and complications is a very academic one, and perhaps should not be addressed within the scope of this research. If we consider that this research is going to be applied to dentists, maybe we should just use the term ‘manifestations’ and group them all together.” In another comment made about question 5, expert 1 suggested, “I think it would be more appropriate to group it (manifestations and complications) all under a single name, since there is no consensus in the literature about what oral manifestations are and what complications are.” Once again, the researchers resorted to the literature and found robust scientific evidence that the terms “repercussions” and “complications” are distinguishable, and that there are studies reporting both repercussions and complications of SCD in the stomatognathic system, hence demonstrating that the terms refer to two conceptually different theoretical domains. An e-mail was sent with feedback to the experts, duly referenced in the literature.

The percentage of agreement regarding questions 7 and 8 of domain IV (“Dentist’s knowledge regarding the dental management of SCD”, questions 7 through 10) ranged from medium to high, whereas the percentage of agreement regarding questions 9 and 10 was high. As regards question 7, “Is there a difference in the dental therapeutic proposal for people with SCD?” one of the experts suggested changing “dental therapeutic proposal” to “dental management,” a suggestion that was promptly accepted by the researchers, since they agreed with the expert’s argument. Regarding question 8, “Do you consider that a person with SCD should undergo antibiotic prophylaxis?” one of the experts said that there was no need for antibiotic prophylaxis for dental procedures in patients with SCD. After revision, this question was kept unchanged, since scientific literature recommends performing antibiotic prophylaxis in certain dental treatments for people with SCD, considering that they are immunosuppressed. Furthermore, in this domain, a question about the use of local anesthetics in dental treatment for patients with SCD was included at the suggestion of experts, because of the relevance of this item, which had not been included in the initial version of the questionnaire.

Finally, although a high level of agreement was observed among the experts’ answers to questions 11, 12 and 13 of domain V (“Dentist’s involvement in an approach to SCD”), some minor improvements were made in terms of the semantics and organization of the answers, and a new question was added at the end of the questionnaire in order to confirm whether the dentist considered that he/she actually knew what SCD was, based on the previous questions. Thus, after evaluation by the expert committee, the final version of the instrument had 16 questions (Figure 1).

**Figure 4:**
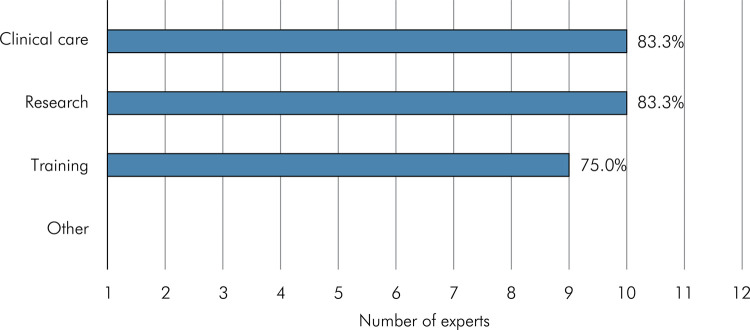
Rates of consensus among experts regarding application options for the instrument.

Ten experts answered the question about the applicability of the instrument. There was 83.3% consensus among experts regarding its applicability in clinical care and research, and 75% consensus relative to its applicability in educational programs. No expert checked the “other” option. Figure 4 shows the percentages of consensus among experts as regards application options for the instrument.

**Figure 5:**
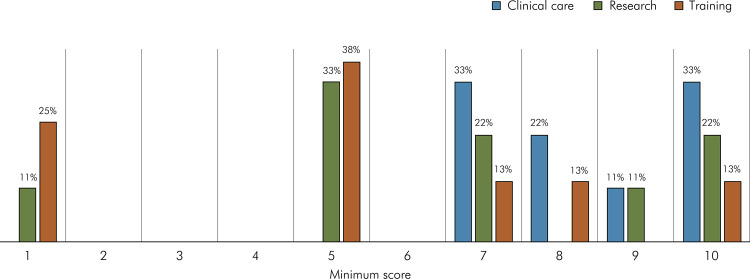
Rates of consensus among experts regarding the minimum scores, on a scale of 1 to 10 that should be achieved to characterize the necessary knowledge for each of the instrument’s applications.

The final two questions the experts were asked about the instrument were “Should there be different minimum scores for the different application options in the questionnaire?” and “Should the weight of any of the items or conceptual domains of the instrument be increased?” Figure 5 shows that the experts chose different minimum scores for the different instrument application options. The initial and final versions of the instrument may be visualized in Figure 1.

## Discussion

The instrument developed to assess the knowledge and involvement of dentists in a dental approach to SCD was evaluated by a committee of experts from the 5 regions of Brazil, showing evidence of the validity of the instrument content for dentists with different backgrounds, with respect to the perspectives of “Knowledge,” “Attitudes,” and “Practices” of dentists in relation to SCD. Thus, our expectation was that not only should the instrument fill a knowledge gap, but that it would also be applied in research, teaching, clinical care. Moreover, the study focused on the instrument applicability both in monitoring the status of the knowledge held by professionals working in health services, and for evaluating the effectiveness of educational interventions, including those in the field of clinical research on the oral health of SCD patients.

The concepts of oral manifestations and oral complications of SCD, and of the dental management of people with the disease were those in which there was greater disagreement among experts. This conceptual framework was directly related to the practice of dentists, and to the design of an instrument about their knowledge, attitudes, and practices related to SCD. In this sense, the literature related to this topic still lacks studies with high levels of scientific evidence, capable of contributing to a high level of recommendation^22^ to support an appropriate dental approach to the disease. This may explain the lack of systematization of terminology, and the incorrect use of words such as “repercussions,” “manifestations,” and “complications,” which are conceptually distinguishable, as though they were synonyms.^9,22-26^The most common orofacial repercussions of SCD are mucosal pallor, dental hypocalcifications (opacities and hypoplasia), pulpal calcifications, avascular pulp necrosis, malocclusion, Osteoporosis and osteosclerosis, increased medullary space, maxillary hyperplasia, craniofacial bone changes, mandibular condyle head necrosis and temporo-mandibular dysfunction. As complications, there are increased risk of infections, mandibular osteomyelitis, orofacial pain, idiopathic facial edema and mentonian nerve neuropathy.

The concept of SCD refers to a set of hemoglobinopathies characterized by a higher frequency of the Hb S mutation. Therefore, SCDs include sickle-cell anemia (Hb SS genotype), SC hemoglobinopathy (Hb SC genotype), SD hemoglobinopathy (Hb SD genotype), and S Beta Thalassemia hemoglobinopathy (Hb S Beta Thalassemia genotype), among others. Despite the genotypic differences among these conditions, their clinical spectrum and the therapeutic approach to them are very similar. It should be noted, however, that sickle-cell anemia occurs more frequently, and progresses with a greater number of complications. Therefore, there are specificities inherent to each genetic profile, and dentists need to know how to identify these profiles, e.g., the laboratory parameters of hematological tests.

When addressing the issue of local anesthetics, the experts drew attention to a relevant issue, namely that there are specific types of anesthetics and vasoconstrictors^27^according to the systemic condition of patients, such as the types specifically for people with SCD. As a first choice, the authors recommended the use of lidocaine with epinephrine, and as a second choice, articaine. This issue had not been covered in the initial version of the instrument, and was incorporated into it, based on the experts’ suggestions. It is noteworthy that the American Academy of Pediatric Dentistry recommends the use of antibiotic prophylaxis^28,29^ for invasive procedures requiring an anesthetic block in the oral cavity to control pain and anxiety, since patients with SCD are immunosuppressed.

Since the instrument is not capable of measuring the variables required for evaluating the involvement of dentists and their knowledge on the subject of SCD, caution is warranted. Hence, consistency among the questions is more important than whether the dentists were able to provide the right or wrong answers.

The contribution made by validating the instrument was to allow a systematic assessment of the personal and technical predisposition of professionals to provide this group of people with adequate care. This, of itself, does not mean that such care will actually be provided. Nevertheless, making the instrument available has been a necessary move towards achieving the desired improvement in care, and towards scientific development with the aim of addressing this gap in providing care. Hence, it should be noted that even after dentists have been evaluated with regard to using the instrument available, these professionals will require training to remedy possible gaps in their knowledge. Likewise, another requirement will be to set up structures and supply the materials needed to support care and scientific development.

The instrument was considered applicable for clinical care, research and training. The need for different minimum cut-off scores for each of the three objectives was also pointed out, considering that the cut-off score for an instrument applied to a professional who already works and has experience with the subject should differ from that attributed to an instrument applied to a general practitioner. The score required for an instrument that assesses the knowledge and involvement of dentists who will provide direct clinical care for people with SCD must be higher. When the objective is training, the cutoff score can be lower than that considered for the other objectives evaluated by the instrument. Furthermore, the domains that refer to SCD manifestations, dental management, and dentist involvement should have greater weight, and differences in the levels of correct answers by the interviewees would indicate different training needs.

To be properly validated, the instrument must specify its application(s).^30^ It is not enough to define the target-audience (in this case, dentists); the intended purpose(s) of measuring the target audience’s knowledge/involvement must also be defined. Applicability of an instrument with the purpose of providing situational diagnoses, before and after an educational intervention, has the same level of importance as that of an instrument providing a situational diagnosis of a dentist that will be recruited for participating in scientific research or for providing care. We understand that a gap in care is closely linked to a gap in scientific evidence which, in turn, is linked to poor professional training. The options for instrument application refer to components of a cycle that needs to be broken, in view of the seriousness of the situation in which the healthcare of SCD patients finds itself. In this sense, the three stated purposes of the instrument are equally necessary, and the consensus among the experts confirmed this equivalence. However, when asked to answer the question about what would be the minimum score that dentists should attain for each of the three applications, the committee responded that the knowledge/involvement demonstrated before educational interventions, required a score lower than that required by the other applications. Once dentists are trained, the knowledge and level of involvement that they acquire could, in principle, be reflected in a greater effort to design and conduct research on the subject, and an even greater effort to provide care. These findings are consistent with those suggested in the literature, about the need for knowing not only the target audience of a given health intervention, but also its goals.^31^ Accordingly, the authors emphasize the importance of thoroughly understanding the applications of the instrument being validated.

The present study contributed to emphasizing the relevance of content validation as a step in the process of developing an instrument for assessing the knowledge and involvement of dentists in the dental approach to SCD. We believe that the pertinence of the concepts and domains present in the initial version of the instrument, and the appropriateness of each item in its final version, in terms of their ability to represent these concepts and domains among the target population, will be able to fill a significant gap in knowledge.^32,33^ Furthermore, this initial effort will ensure that the next validation steps to be carried out in a future study will be substantiated by content, of which the quality of evidence has been assured. As limitations, each question was composed of a detailed explanation of the references used and the researchers´ experience in constructing the item. Moreover, only biological aspects of the major framework for the outcomes of SCD were considered for validation, underestimating the requirement of including a discussion of structured racism related to the oral health outcomes of people with SCD.

## Conclusion

The expert committee showed evidenced of the content validity of the instrument.
